# Clinical features and maternal-fetal outcomes of patients with first-onset acute leukemia during pregnancy: a retrospective study from pregnancy hematological disease referral center

**DOI:** 10.1186/s12884-026-08737-7

**Published:** 2026-02-04

**Authors:** Xue Xu, Mei-ying Liang, Xiao-hui Zhang, Yi-min Zhang, Sheng-long Ye

**Affiliations:** 1 Obstetrics and Gynecology, Peking University People’s Hospital, No. 11, Xizhimen South Street, Xicheng District, Beijing, China; 2 Referral Center of Pregnancy Complicated with Hematological Diseases, Beijing Municipal Health of Commission, Health of Commission, Beijing, China; 3 Hematology, Peking University People’s Hospital, Beijing, China; 4 Pediatrics, Peking University People’s Hospital, Beijing, China

**Keywords:** Pregnancy, First-onset acute leukemia, Maternal-fetal outcome, Prognostic factor, Chemotherapy safety

## Abstract

**Objective:**

This study aimed to characterize the clinical features, maternal-fetal outcomes, and prognostic factors of pregnant patients with first-onset acute leukemia (AL) at a specialized referral center in China.

**Methods:**

A retrospective descriptive study was conducted on 53 pregnant patients diagnosed with de novo AL and treated at Peking University People’s Hospital between January 2010 and January 2024. Clinical data, including maternal baseline characteristics, treatment protocols, pregnancy outcomes, and long-term maternal/neonatal follow-up results, were collected and analyzed. Survival outcomes were estimated using Kaplan-Meier curves, and prognostic factors for maternal mortality were identified via Cox regression analysis.

**Results:**

Of the 53 patients, 40 (75.5%) had acute myeloid leukemia (AML) and 13 (24.5%) had acute lymphoblastic leukemia (ALL); AL onset occurred in the first (*n* = 12, 22.6%), second (*n* = 28, 52.8%), and third (*n* = 13, 24.6%) trimesters. The most common initial symptoms were oral manifestations (34.0%, e.g., gingival bleeding/hyperplasia), while thrombocytopenia (94.3%) and anemia (86.8%) were the most prevalent peripheral blood abnormalities. 23 (43.4%) patients reached the peripartum period (≥ 28 weeks), with 56.5% preterm births and 13.0% fetal growth restriction (FGR). Gestational age at AL onset was the only factor significantly associated with peripartum progression (OR = 1.642, *p* < 0.001). Seven patients received antenatal induction chemotherapy, all achieving live births. Long-term follow-up (median: 4.4 years) of 17/22 live-born children showed no growth restriction or neurodevelopmental delays. The 5-year overall survival (OS) and disease-free survival (DFS) rates for mothers were 50.9% and 41.5%, respectively. Cox regression identified disseminated intravascular coagulation (DIC, HR = 5.274, *p* = 0.022) and leukocytosis (HR = 5.009, *p* = 0.025) as independent risk factors for maternal mortality; chemotherapy delay (> 30 days) was associated with lower complete remission (CR) rates (66.7% vs. 87.1%) and higher mortality (50% vs. 26.7%).

**Conclusion:**

Unexplained thrombocytopenia or oral manifestations are key early indicators for targeted AL screening in pregnant patients. Gestational age at AL onset was the only factor significantly associated with peripartum progression. Disseminated intravascular coagulation and leukocytosis were independent risk factors for maternal mortality. Among 17 live-born children with a median follow-up of 4.4 years, no growth restriction or neurodevelopmental delays were observed, including 6 infants exposed to antenatal ATRA-based chemotherapy.

## Background

Acute leukemias (AL) occurring during pregnancy pose unique and complex clinical challenges in the fields of diagnosis and treatment. Currently, the exact incidence of pregnancy-associated acute leukemia remains incompletely defined. Available epidemiological data estimate its incidence at approximately 1.6 to 2.3 cases per 100,000 pregnancies [1–3]. The incidence of such diseases has increased 2.7% per annum in pregnancy-associated cancer in 1994 ~ 2013. This change has been attributed to advancing maternal age, improved diagnostic techniques, and increased health-system engagement [4, 5]. Reported cases exhibit a distinct disease subtype distribution: roughly two-thirds are acute myeloid leukemia (AML), while the remaining one-third are acute lymphoblastic leukemia (ALL) [6, 7]. Significantly, certain clinical manifestations of this disease, such as fatigue, nausea, and elevated white blood cell counts, closely mimic normal physiological changes during pregnancy. This similarity substantially complicates early disease recognition and diagnosis. Additionally, pregnancy-associated acute leukemia is characterized by high malignancy and rapid progression, markedly increasing the risk of severe maternal complications, including disseminated intravascular coagulation (DIC), active bleeding, severe infections, and thrombotic events [2]. These complications not only threaten maternal life but also pose significant challenges to fetal outcomes. For high-risk pregnancies complicated by hematological diseases like AL, specific laboratory tests can facilitate early detection of coagulation abnormalities—for instance, D-dimer, which reflects fibrin degradation, and fibrin monomer, a key marker of early coagulation activation. These indicators have been validated in clinical practice to effectively identify subtle coagulation disorders in high-risk pregnant populations, thereby providing a basis for timely intervention [8]. Given the aggressive biological behavior of acute leukemia, clinical treatment cannot be indefinitely delayed. Thus, a delicate clinical balance must be established between the potential risks of high-dose chemotherapy to the mother and fetus, and the adverse consequences of treatment delay on maternal disease progression.​.

To address these clinical challenges, the Hematological Malignancy Task Force of the British Committee for Standards in Hematology has published specialized clinical guidelines focusing on the diagnostic pathways and management strategies for pregnancy-associated AML [9]. However, high-quality clinical data for this specific population remain scarce globally. There is a particular lack of systematic research evidence regarding pregnancy outcomes and long-term maternal and fetal prognoses, significantly hindering the optimization and dissemination of clinical guidelines.​.

As a leading referral center for pregnancy-related hematological disorders in China, our hospital initiated this retrospective study leveraging its clinical resource advantages. The core objective of this study is to comprehensively describe the clinical features. Through in-depth analysis of key data, including clinical characteristics, occurrence of pregnancy complications, and long-term maternal and fetal outcomes, we aim to generate systematic clinical evidence. As the world’s most populous country, China has a relatively large number of patients with pregnancy-complicated acute leukemia. The unique clinical features and maternal-fetal outcome data from this population hold significant international reference value. Therefore, this single-center, retrospective descriptive study aimed to comprehensively characterize the clinical and laboratory features of patients with first-onset acute leukemia during pregnancy, to evaluate both immediate and long-term maternal and fetal outcomes, and to identify potential prognostic factors influencing maternal survival.

## Method

This retrospective descriptive study included patients who were diagnosed with acute leukemia (AL) during pregnancy and received treatment at Peking University People’s Hospital between January 2010 and January 2024. The eligibility criteria were defined as follows: (1) age ranging from 18 to 40 years; (2) de novo diagnosis of AL during pregnancy; and (3) complete clinical documentation available. Patients with pregnancies occurring during or after AL treatment were excluded from the study.

All enrolled patients were diagnosed in accordance with the World Health Organization (WHO) 2016 Classification of Hematopoietic and Lymphoid Tumors [10], based on a comprehensive assessment including morphological, immunological, cytogenetic, and molecular profiling (MICM profiling). This study was approved by the Ethics Committee of Peking University People’s Hospital. Given its retrospective design, a waiver of informed consent was granted by the ethics committee.

### Data collection

#### Maternal and pregnancy-related data

Collected variables included maternal age, obstetric history (e.g., parity, previous adverse pregnancy outcomes), pre-pregnancy body weight, height, gestational age at AL onset, gestational age at abortion or delivery, method of labor (e.g., vaginal delivery, cesarean section), maternal complications (e.g., infection, hemorrhage, disseminated intravascular coagulation), fetal complications (e.g., fetal growth restriction, preterm birth), and serial complete blood count (CBC) results during the entire pregnancy.

Peripartum progression is formally defined as: pregnant patients with first-onset AL who maintain an ongoing pregnancy and reach ≥ 28 weeks of gestation rather than therapeutic abortion occurring due to disease.

Postpartum hemorrhage (PPH) was defined according to international obstetric clinical practice guidelines and stratified by delivery or pregnancy termination mode. For the purpose of this study, the following terms are uniformly defined and used: (1) Vaginal delivery: blood loss ≥ 500 mL within 24 h after delivery; (2) Cesarean section: blood loss ≥ 1000 mL within 24 h after delivery; (3) Therapeutic abortion: pregnancy termination with blood loss ≥ 300 mL during the procedure or within 24 h after the procedure.

#### Acute leukemia related data

This dataset comprised initial clinical symptoms at prenatal AL diagnosis (e.g., fatigue, fever, bleeding), findings of prenatal examinations (e.g., abnormal blood cell counts on routine prenatal screening), AL subtype (AML or ALL) and detailed immunophenotypic, cytogenetic, and molecular characteristics, timing of AL diagnosis, and specifics of anti-leukemic treatment (e.g., chemotherapy regimens, drug dosages, treatment delays due to pregnancy). For patients receiving chemotherapy, complete remission (CR) was defined as follows: platelet count ≥ 100 × 10⁹/L, absolute neutrophil count ≥ 1.5 × 10⁹/L, bone marrow blast percentage ≤ 5%, and no evidence of extramedullary leukemia infiltration.

#### Reference for leukocyte count thresholds

Baseline leukocyte counts exhibit dynamic changes throughout pregnancy and the postpartum period. To establish thresholds for defining abnormal leukocyte count elevations or reductions in this study, we referenced a large-scale observational study conducted in Turkey, which analyzed leukocyte count fluctuations across all trimesters of pregnancy and the early postpartum period in 40,325 healthy pregnant women [11].

#### Treatment protocols

Induction chemotherapy for AML (including acute promyelocytic leukemia [APL]) and ALL followed institutional protocols for pregnancy-associated hematological disorders. Given the complexity of balancing maternal anti-leukemic efficacy and fetal safety, all specific treatment regimens (including the selection of chemotherapeutic agents, dosage adjustments, and combination strategies) were formulated through multidisciplinary consultations led by hematologists specialized in pregnancy-associated hematological diseases.

#### Follow-up protocols

Surviving patients were followed up until Dec. 31, 2024, whereas follow-up for deceased patients was censored at the date of death. Follow-up was conducted via three approaches: telephone interviews, outpatient clinical visits, and review of electronic medical records. Two key survival endpoints were defined:

Overall Survival (OS): Calculated from the date of AL diagnosis to the date of death from any cause or the date of the last follow-up visit.

Disease-Free Survival (DFS): Defined as the time from the first documentation of CR to the date of confirmed disease relapse or death from any cause.

Neonatal follow-up was jointly performed by obstetricians and pediatricians, with the follow-up period concluding on Dec. 31, 2024. Follow-up for live-born children included assessments of physical growth and neurodevelopment. Height and weight of the children were measured. Referring to the nationally representative growth reference curves and anthropometric standards in the *Chinese Child Growth Standards* [12], children with height or weight below 2 standard deviations were considered to have growth retardation. Neurodevelopmental evaluation was performed using two culturally validated and standardized Chinese versions of validated instruments: the *Ages and Stages Questionnaire*,* Third Edition* [13] (ASQ-3; applicable for 1–65 months) and its complementary module, the *Ages and Stages Questionnaire: Social–Emotional* [14] (ASQ: SE; applicable for 3–65 months). Domain-specific raw scores underwent norm-referenced standardization were categorized as typical development (above the cutoff) or referral zone (below two standard deviations).

### Statistical analysis

All statistical analyses were performed using SPSS software (Version 26.0; IBM Corp., Armonk, NY, USA). Continuous data were first tested for normality using the Shapiro-Wilk test. For normally distributed continuous data, results are presented as mean±standard deviation (SD), and between-group comparisons were conducted using independent samples t-tests (for two groups) or one-way analysis of variance (ANOVA) (for three groups). For non-normally distributed continuous data, results are expressed as median (interquartile range [IQR]), and between-group comparisons were performed using the Wilcoxon rank-sum test. Categorical data are presented as n (percentage), and between-group comparisons were analyzed using the Fisher’s exact test.​.

Kaplan-Meier survival curves were generated to estimate OS and DFS rates, and between-group differences in survival were compared using the log-rank test. Cox proportional hazards regression analysis was employed to identify factors influencing maternal mortality after verifying the proportional hazards assumption via the Schoenfeld residual test. Potential covariates were pre-specified based on clinical relevance and univariate analysis results. Hazard ratios (HR) and their corresponding 95% confidence intervals (CI) were calculated to quantify the association between each covariate and mortality risk. Given the small number of outcome events relative to the number of covariates, this Cox regression analysis was presented as exploratory, aiming to generate hypotheses regarding potential prognostic factors rather than confirm definitive associations. A two-tailed P-value < 0.05 was considered statistically significant.

## Result

### Maternal baseline characteristics, clinical manifestations and pregnancy outcomes

**Fig. 1:**
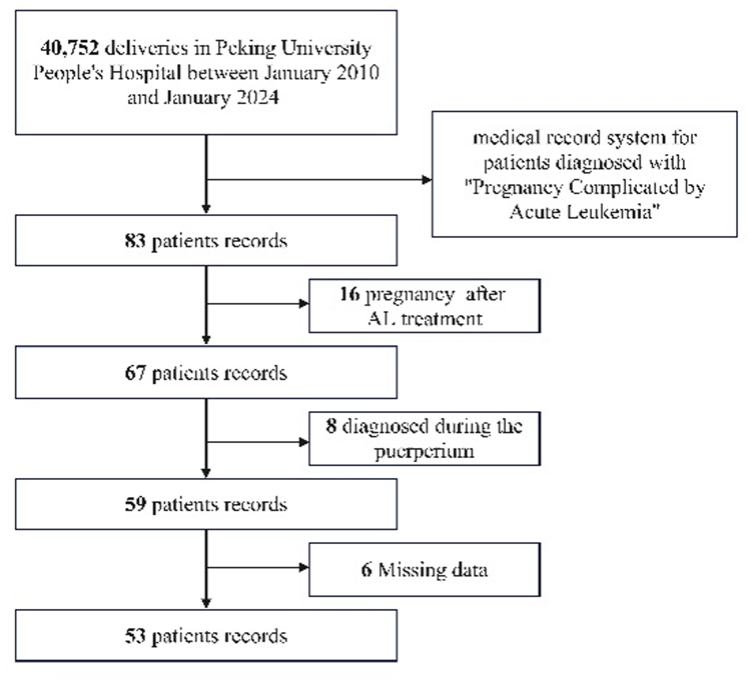
Case selection flowchart

In the present study, we consecutively included 53 pregnant patients who were diagnosed with de novo AL, including 40 cases of AML and 13 cases of ALL. Based on the timing of AL onset relative to gestational stage, 12 patients developed the disease in the first trimester, 28 in the second trimester, and 13 in the third trimester. The detailed process of patient screening, exclusion, and final enrollment—conducted in accordance with the STROBE reporting guidelines for observational studies—is illustrated in Fig. 1.

Detailed demographics and general clinical characteristics of patients stratified by these gestational onset subgroups are summarized in Table 1. Statistical analyses demonstrated no significant differences in key clinical parameters-including maternal age, leukemia subtype, and gravidity-among patients with AL onset in different trimesters of pregnancy.

**Table 1:** Characteristics of patients with acute leukemia onset at various stages of pregnancy

Characteristics	Trimester in which AL was diagnosed			*P* value
	First trimester	Second trimester	Third trimester	
Case number (n)	12	28	13	
Median age (y)	30.9(± 5.0)	29.7(± 5.7)	26.9(± 5.1)	0.166
Type of leukemia(n,%)				
AML	9(75)	22(78.6)	9(69.2)	0.911
ALL	3(25)	6(21.4)	4(30.8)	
Previous pregnancy(n,%)				
Yes	5(41.7)	11(39.3)	5(38.5)	1.000
Pregnancy outcomes (n,%)				
Therapeutic abortion	11(91.7)	17(60.7)	0	˂0.001
Caesarean sections	-	5(17.8)	8(61.5)	-
Vaginal deliveries	-	5(17.8)	4(30.8)	-
Death	1(8.3)	1(3.7)	1(7.7)	0.783
Response to induction chemotherapy (n,%)				
Complete remission	8(66.7)	22(78.6)	5(38.5)	0.522
Recurrence	1(8.3)	8(28.6)	1(8.7)	0.071
Maternal and neonatal outcomes(n,%)				
Premature birth	-	5(50.0)^†^	8(61.5)	-
Preeclampsia	-	1(10)^†^	1(7.7)	-
DIC	4(33.3)	2(7.1)	1(7.7)	0.106
Postpartum hemorrhage	2(16.7)	5(17.9)	3(23.1)	0.902
FGR	-	3(30)^†^	0	-
Infection	0	3(10.7)	1(7.7)	0.398
Maternal death*	1(8.3)	1(3.7)	1(7.7)	0.783

Continuous data for maternal age were analyzed using one-way analysis of variance (ANOVA). All other categorical variables were compared using Fisher’s exact test

*AML *Acute myeloid leukemia, *ALL *Acute lymphoblastic leukemia, DIC Disseminated intravascular coagulation, *FGR *Fetal growth restriction

†Denominator excluded patients who failed to reach the peripartum period (*n* = 10)

*Death that occurs within postpartum and before chemotherapy begins

Table 2 summarizes the primary onset symptoms of acute leukemia in pregnant patients, with oral manifestations emerging as the most prevalent—observed in 34% of cases—including gingival bleeding, hyperplasia, swelling, and pain. In initial abnormal peripheral blood tests, the distribution of white blood cell counts was similarly distributed across three categories: elevated, normal, or reduced. The median leukocyte count was 7 × 10⁹/L, with a corresponding range of 0.95–145 × 10⁹/L. Conversely, anemia and thrombocytopenia exhibited notably high incidences, affecting 86.8% and 94.3% of patients, respectively. A critical observation is that in 5 cases, the diagnosis of acute leukemia was only confirmed following delayed identification of thrombocytopenia, despite the absence of overt clinical symptoms throughout the early course.

**Table 2:** Early symptoms and first occurrence of abnormal peripheral blood examination results of patients with acute leukemia during pregnancy

Clinical Symptoms and Peripheral Blood Examination at Onset	Case (*n*, %)
Asymptomatic	19 (35.8)
Gingival bleeding or hyperplasia, swelling and pain	18 (34.0)
Weakness	9 (17.0)
Bleeding points or nosebleeds	8(15.1)
Pallor	2 (3.8)
Fever	7 (13.2)
Peripheral Blood counts	
Leukocytosis (>17.5 × 10^9^/L)	17(32.1)
Leukocytopenia (<4.5 × 10^9^/L)	23(43.4)
Normal leukocytes	13(24.5)
Anemia (<110 g/L)	46(86.8)
Thrombocytopenia (<100 × 10^9^/L)	50(94.3)

For patients with disease onset in the first trimester of pregnancy, all underwent therapeutic abortion. Among those with second-trimester onset, the rate was 60.7%. A total of 23 patients progressed to the peripartum period (≥ 28 weeks of gestation), including 1 case of stillbirth and 22 live births. The mean gestational age at delivery was 34.1 ± 3.1 weeks; of these deliveries, 56.5% (13/23) were classified as preterm, and 13.0% (3/23) were complicated by fetal growth restriction (FGR).

Intergroup logistic regression analysis revealed that the probability of patients reaching the peripartum period was significantly associated with gestational age at disease onset (*p* < 0.001; odds ratio [OR] = 1.642). In contrast, no significant association was observed between this probability and the following factors: maternal age, parity, presence of disseminated intravascular coagulation (DIC), or peripheral blood parameters (white blood cell count, hemoglobin level, platelet count) (all *p* > 0.05).

### Antenatal chemotherapy exposure and neonatal outcomes

Among the study participants, seven pregnant patients underwent antenatal induction chemotherapy. All seven cases were diagnosed with acute promyelocytic leukemia (APL) and treated with all-trans retinoic acid (ATRA)-based induction therapy; regimens were individualized by adding arsenic trioxide (ATO) or anthracyclines following hematologist consultation. Of these patients, 3 received combination therapy: 2 were given ATRA plus daunorubicin, and 1 received the triple regimen of ATRA, daunorubicin and ATO. Notably, all seven patients achieved live births, with a median gestational age at delivery of 37.3 weeks; among these deliveries, three were preterm, and one was complicated by fetal growth restriction (FGR).

For the 22 live births, neonatal outcomes were compared between the chemotherapy-exposed group and the non-exposed group. As shown in Table 3, the two groups exhibited no statistically significant differences in key neonatal outcomes, including gestational age at birth, birth weight, preterm birth rate, and neonatal intensive care unit (NICU) admission rate. Long-term follow-up was conducted for 17 of 22 live-born children (6 in the chemotherapy-exposed group, 11 in the non-exposed group). The median follow-up age of these children at last follow-up was 4.4 years, with a range of 9 months to 10.3 years. There was no growth restriction and neurodevelopmental delays were observed in this small sample.

**Table 3:** Fetal outcomes in pregnancies with and without antenatal chemotherapy exposure

Outcome Index	Exposed group *N* = 7	non-exposed group *N* = 15	*P* value
Gestational age at delivery (Median[Q1,Q3], weeks)	37.3(36.0,38.4)	34.4(32.2,37.0)	0.072
Premature birth (n,%)	3(42.9)	10(66.7)	0.376
FGR (n,%)	1(14.3)	2(13.3)	1.000
Birth weight (mean ± SD, g)	2832 ± 781	2270 ± 488	0.051
NICU admission (n,%)	2(28.6)	10(66.7)	0.172

Gestational age at delivery was analyzed using the Wilcoxon rank-sum test; Birth weight was using the independent samples t-test. All other categorical variables were analyzed using Fisher’s exact test. FGR=fetal growth restriction; NICU= neonatal intensive care unit; SD= standard deviation

### Maternal long-term outcome

Among the 53 patients, CR rate was 66.0% (35/53) following induction chemotherapy. Subsequently, 27 patients continued with consolidation chemotherapy, while the remaining patients underwent hematopoietic stem cell transplantation (HSCT). During the follow-up period, 10 patients experienced relapse.

As of the end of the follow-up period, the median follow-up time for the 53 patients was 89.0 ± 10.6 months, with 10 patients lost to follow-up and 16 patients deceased, including 3 who died before they could receive chemotherapy post-induction. Overall, the estimated 5-year overall survival (OS) and disease-free survival (DFS) rates for the 53 patients with pregnancy-associated AL are 50.9% and 41.5%, respectively.

In the present study, after excluding patients with early death (those who died before receiving chemotherapy) and lost to follow-up, a total of 43 patients underwent chemotherapy during labor or postpartum. Among them, 12 patients had chemotherapy delayed for more than 30 days due to various reasons. The complete remission (CR) rates were 66.7% (8/12) in the chemotherapy delay group versus 87.1% (27/31) in the immediate chemotherapy group, and the mortality rates at the end of follow-up were 50% (5/10) versus 26.7% (8/30), respectively.

**Fig. 2:**
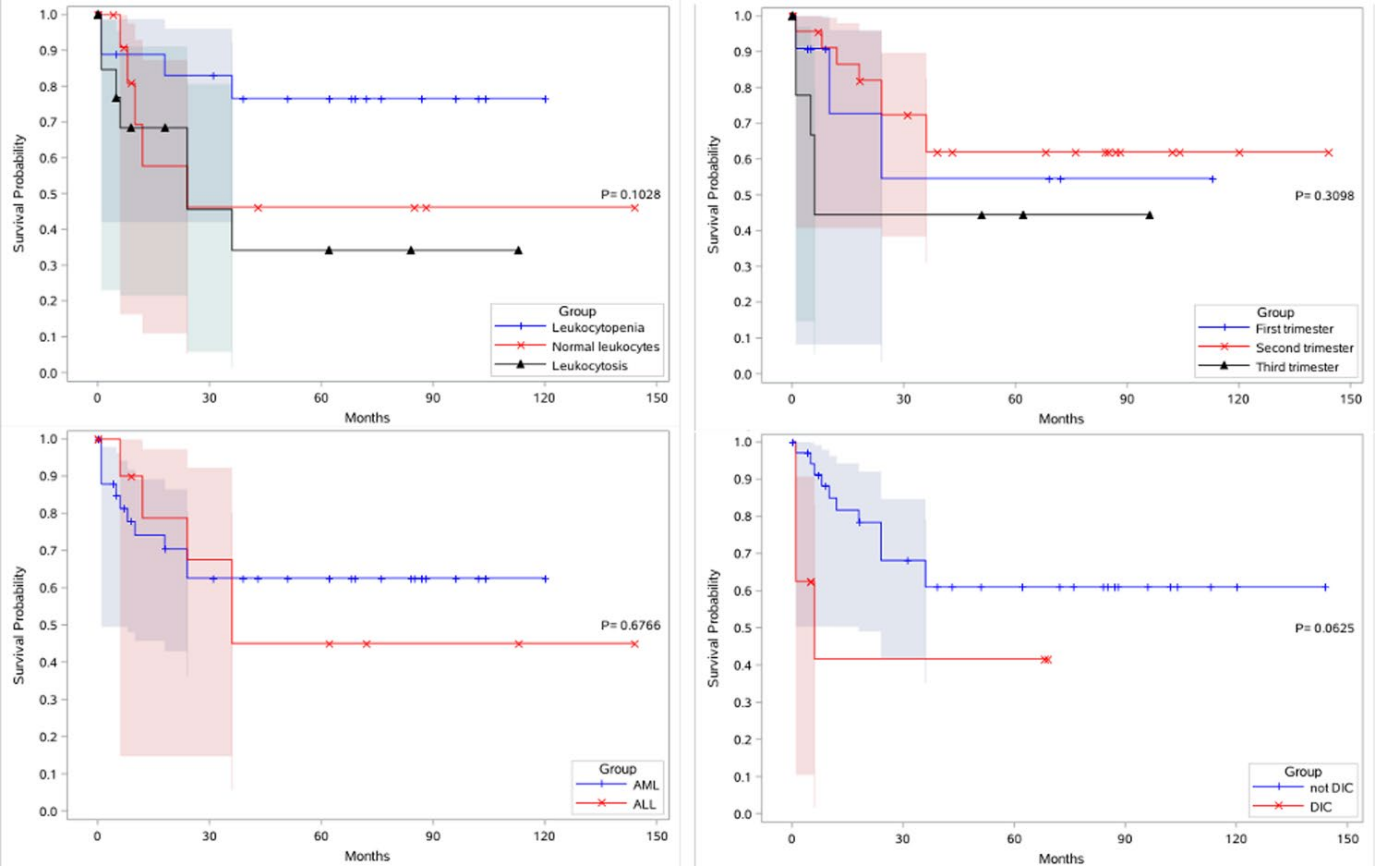
Survival outcomes of 53 pregnant patients with de novo acute leukemia. **A** Survival probability by acute leukemia subtype (AML vs. ALL). **B** Survival probability by trimester of onset (1st vs. 2nd vs. 3rd). **C** Survival probability by leukocyte status (Leukocytopenia vs. Normal vs. Leukocytosis). **D** Survival probability by presence of DIC

Survival curve analysis was performed by comparing factors including leukemia subtypes, gestational stage at disease onset, leukocyte count at diagnosis, and presence of DIC during pregnancy (Fig. 2). It should be clarified that since the focus of this study is on obstetric aspects, we did not conduct relevant prognostic analysis on key hematological indicators; the classification of AML and ALL was only performed as one of the contextual background data. The results showed no statistically significant differences among the four factors in the log-rank test. However, log-rank trend test revealed a significant linear association between leukocyte count and mortality risk (*P* = 0.035). Cox regression analysis revealed that the presence of DIC during pregnancy or leukocytosis was an independent high-risk factor for death (Table 4).

**Table 4:** Univariate Cox regression analysis of factors associated with mortality in pregnant patients with AL

Variable	HR	95%CI	*P* value
AML	1.091	0.293–4.068	0.836
Trimester at disease onset			
First trimester	0.374	0.086–1.623	0.189
Second trimester	0.444	0.134–1.472	0.184
DIC	5.009	1.264–19.838	0.022
Leukocyte status			
Leukocytosis	5.274	1.235–22.530	0.025
Normal leukocytes	3.861	0.962–15.494	0.057

*HR *Hazard Ratio, 95% *CI*  95% Confidence Interval, *AML *Acute myeloid leukemia, *DIC *Disseminated intravascular coagulation

## Discussion

Consistent with previous studies, 75.5% of our patients had AML and 24.5% had ALL. Additionally, 77.3% were diagnosed in the second or third trimester, and no significant differences in AL subtypes were observed across pregnancy stages.

Pregnancy is a unique physiological state, and some of its manifestations may mimic hematologic malignancies. During the early stages, differentiating pregnancy-related physiological changes from the early signs of acute malignancy is extremely difficult [3, 6]. However, from our research, it can still be found that gingival bleeding or hyperplasia is the most common clinical manifestation in acute leukemia during pregnancy. Physiological dynamic changes in white blood cells during pregnancy may interfere with the identification of leukemia. A large-scale study involving 40,325 pregnant individuals, encompassing 82,786 complete blood count assessments conducted between the 6th week of gestation, through the 41st week, and into the early postpartum period, provides critical reference ranges. The data reveal that the lowest leukocyte value (3rd percentile) occurs in the first trimester, at 4.5 × 10⁹/L, while the highest value (99th percentile) peaks in the third trimester at 17.5 × 10⁹/L [11].​Notably, our findings indicate that approximately one-quarter of pregnant patients with leukemia present with normal leukocyte counts during the early onset stage despite these physiological ranges. In contrast, almost all such patients exhibit concurrent thrombocytopenia. This observation underscores the clinical significance of thrombocytopenia in pregnancy: for cases of newly developed thrombocytopenia during gestation, peripheral blood smear examination should be regarded as a necessary screening modality to facilitate timely identification of underlying hematological malignancies such as leukemia. The diagnostic criteria for AML are the same in pregnant patient as in non-pregnant women. These criteria are defined in the World Health Organization (WHO) classification of the myeloid neoplasms.

The timing of AL diagnosis during pregnancy—specifically, the gestational trimester—plays a crucial role in determining pregnancy outcomes. In the present study, all patients diagnosed in the first trimester opted for therapeutic abortion, while over half of those diagnosed in the second trimester underwent labour induction. The overall pregnancy loss rate reached 58.5%, which surpasses the rates observed in cases of solid tumours and some retrospective cohort studies [15]. Progression to the peripartum period ≥ 28 weeks of gestation is the threshold for fetal viability. It directly relates to neonatal survival which requires coordinated hematological and obstetric management, addressing a key clinical challenge. Regression analysis revealed that the primary factor contributing to pregnancy loss was the gestational age at diagnosis, with no significant association identified with concurrent infections, disseminated intravascular coagulation (DIC), or blood cell counts. The majority of pregnancy losses were attributed to therapeutic abortion due to disease considerations, with the incidence of intrauterine fetal death (IUFD) being only 5.7%. The high rate of therapeutic abortion in the first and second trimesters likely reflects concerns about teratogenic and neurodevelopmental risks. Moreover, the aggressive nature of AL often precludes delaying treatment, making it impractical to allow the pregnancy to progress beyond the first trimester or reach full term.

Importantly, postpartum hemorrhage in this study is not merely an isolated complication but a precursor to more severe events. Specifically, the combination of AL-related coagulopathy and pregnancy-induced hypercoagulability increases the risk of pulmonary embolism and subsequent cardiac arrest [16]. Maternal morbidity and obstetric complications were notably more prevalent among women with hematological malignancy during pregnancy than in the reference population [2, 3]. We observed a significant incidence of preterm birth in this study, with induced prematurity accounting for half of these cases, likely to facilitate the initiation or continuation of chemotherapy. The underlying causes of spontaneous preterm births in the remaining cases remain unclear. Furthermore, the rates of thrombosis, DIC, infections, and caesarean section among patients with acute leukemia were significantly higher than those in the general population, which is consistent with previous reports [2, 17].

To date, no valid evidence from domestic or international studies has confirmed a definitive association between pregnancy and hematologic malignancies. Current evidence fails to demonstrate that the complete remission (CR) rate of acute leukemia during pregnancy is significantly lower than that in non-pregnant patients. Multiple studies have shown that pregnant patients with acute leukemia can still achieve a relatively high CR rate after induction chemotherapy. In one cohort study involving 23 patients, 78% (18/23) achieved complete remission [18]; in another study with 21 patients, the CR rate among pregnant patients with acute leukemia who received chemotherapy was 86% (19/22) [19]. In the present study, the CR rate is slightly lower than the levels reported in international clinical studies. However, considering the retrospective nature of this study and its relatively long time span, there may be a time trend bias.

However, a high CR rate is not accompanied by a high survival rate. The overall mortality rate of pregnancy-associated AL has been reported to range from 33% to 78% across different studies. Another study on 52 cases of pregnancy-associated AL found that the median survival time of pregnant women who received chemotherapy was only 16 months [20].

This study is the first to conduct statistical analysis on the factors influencing the long-term maternal prognosis of AL during pregnancy. The results showed that patients with elevated white blood cell counts during pregnancy had a poorer prognosis. High white blood cell counts have been associated with adverse prognoses in previous studies, and acute myeloid leukemia (AML) during pregnancy is more likely to present with high white blood cell counts [21]. The combination of hypercoagulable state and leukostasis gives acute leukemia during pregnancy more aggressive biological characteristics.

Patients with acute leukemia during pregnancy (especially acute promyelocytic leukemia [APL]) are prone to complicated disseminated intravascular coagulation (DIC), and DIC is a key factor leading to early death (ED). Pregnancy itself can induce DIC due to obstetric complications (such as pre-eclampsia and placental abruption), and if combined with acute leukemia, their synergistic effect will further worsen the prognosis. A study showed that women with pregnancy-associated acute leukemia are more likely to develop DIC (with a significantly increased adjusted risk). Early diagnosis and targeted treatment are crucial to improving the prognosis.

Most cytotoxic agents have a molecular weight between 250 and 400 kDa, meaning virtually all can cross the placenta to reach the fetus. A study of 84 children born to mothers treated with chemotherapy for hematological malignancies found no congenital, neurological, immunological, or psychological abnormalities [22]. Although acute myocardial dysfunction can appear in the fetus during pregnancy with anthracyclines [23, 24]. In a few cases, secondary malignancies, growth retardation, intellectual impairment, and reduced fertility have been reported [25, 26]. I In contrast, the ATRA-based regimens used in our study minimized fetal exposure to cytotoxic agents. This may explain why neonatal outcomes were more favorable in the chemotherapy-exposed group compared to the non-exposed group. During postnatal follow-up, no growth or neurodevelopmental delays were identified in the 17 live-born children assessed. Nevertheless, the long-term effects of in utero ATRA exposure (and limited cytotoxic exposure) require further investigation.

### Strengths and limitations

Our study has several notable strengths. To our knowledge, this represents one of the largest single-center cohorts to date specifically investigating first-onset acute leukemia during pregnancy, providing robust data from a major national referral center. The comprehensive and standardized collection of data allowed for a detailed analysis of clinical presentation, treatment protocols, and a broad range of outcomes, including long-term maternal survival and neonatal growth and neurodevelopment. Furthermore, our analysis identified key prognostic factors, such as leukocytosis and disseminated intravascular coagulation, offering valuable insights for risk stratification.

However, the findings must be interpreted in the context of the study’s limitations. The retrospective design is inherently susceptible to selection bias and unmeasured confounding factors, despite our efforts to include all eligible patients. As a study conducted at a tertiary referral center, our patient population may represent more severe or complex cases, potentially limiting the generalizability of our results to non-specialized settings. It is important to acknowledge that this study did not include established hematological prognostic factors (e.g., European Leukemia Net risk classification) in the prognostic analysis, which was a deliberate design choice aligned with the study’s core focus and target audience. Compared with non-pregnant AL patients, pregnant individuals face distinct clinical challenges and our aim was to identify obstetric-specific predictors and outcomes that guide perinatal clinical decision-making. We recognize the value of integrating classic hematological prognostic indices, and our collaborative hematology team is conducting a follow-up study to compare hematological markers between pregnant and non-pregnant AL patients, which will provide complementary insights into this special population. Although the neonatal follow-up rate was high, the sample size for the chemotherapy-exposed group remained small, limiting the statistical power to detect subtle adverse effects. Crucially, the follow-up period was insufficient to evaluate long-term sequelae in children, such as secondary malignancies, fertility issues, or very late-onset neurodevelopmental deficits, which require decades of observation.

## Conclusion

In summary, this study provides key clinical insights for the management of first-onset acute leukemia (AL) during pregnancy. Targeted AL screening should be prioritized for pregnant patients with unexplained thrombocytopenia or oral manifestations, as these were the most common early indicators of the disease identified in our analysis. Gestational age at AL onset was the only factor significantly associated with peripartum progression (OR = 1.642, *p* < 0.001), playing a pivotal role in guiding pregnancy management decisions. Additionally, exploratory Cox regression analysis identified disseminated intravascular coagulation (DIC, HR = 5.009, *p* = 0.022) and leukocytosis (HR = 5.274, *p* = 0.025) as independent high-risk factors for maternal mortality.

## Data Availability

The datasets generated and/or analyzed during the current study are available from the corresponding author upon reasonable request.

## Abbreviations

AL: Acute Leukemia
AML: Acute Myeloid Leukemia
ALL: Acute Lymphoblastic Leukemia
FGR: Fetal Growth Restriction
DIC: Disseminated Intravascular Coagulation
OS: Overall Survival
DFS: Disease-Free Survival
CR: Complete Remission
HR: Hazard Ratio
CI: Confidence Interval
MICM: Morphological, Immunological, Cytogenetic, and Molecular Profiling
APL: Acute Promyelocytic Leukemia
ATRA: All-Trans Retinoic Acid
HSCT: Hematopoietic Stem Cell Transplantation
CBC: Complete Blood Count
NICU: Neonatal Intensive Care Unit
IQR: Interquartile Range
SD: Standard Deviation
ANOVA: Analysis of Variance
ASQ-3: Ages and Stages Questionnaire, Third Edition
ASQ:SE: Ages and Stages Questionnaire: Social–Emotional
WHO: World Health Organization
SPSS: Statistical Package for the Social Sciences
IUFD: Intrauterine Fetal Death
ED: Early Death
